# The demography of COVID-19 deaths database, a gateway to well-documented international data

**DOI:** 10.1038/s41597-022-01191-y

**Published:** 2022-03-22

**Authors:** Arianna Caporali, Jenny Garcia, Étienne Couppié, Svitlana Poniakina, Magali Barbieri, Florian Bonnet, Carlo Giovanni Camarda, Emmanuelle Cambois, Iris Hourani, Daria Korotkova, France Meslé, Olga Penina, Jean-Marie Robine, Markus Sauerberg, Catalina Torres

**Affiliations:** 1 French Institute for Demographic Studies (INED), 9 cours des Humanités, CS 50004, 93322 Aubervilliers, Cedex France; 2 Department of Demography, University of California-Berkeley, 2234 Piedmont Avenue, Berkeley, CA 94720-2120 United States; 3 Department for social and demographic statistics, Ptoukha Institute for Demography and Social Studies of the National Academy of Sciences of Ukraine, Shevchenko blvd., 60, Kyiv, 01032 Ukraine; 4 Department of Social Medicine and Management, Nicolae Testemitanu State University of Medicine and Pharmacy, 194b Stefan cel Mare si Sfant Blvd, Chisinau, MD-2004 Republic of Moldova; 5 National Institute of Health and Medical Research (INSERM), Paris, France; 6 Federal Institute for Population Research, Friedrich-Ebert-Allee 4, 65185 Wiesbaden, Germany; 7 Eco-anthropologie (EA), Muséum national d’Histoire naturelle, CNRS, Université de Paris, Musée de l’Homme 17 place du Trocadéro, 75016 Paris, France

**Keywords:** Social sciences, Infectious diseases

## Abstract

National authorities publish COVID-19 death counts, which are extensively re-circulated and compared; but data are generally poorly sourced and documented. Academics and stakeholders need tools to assess data quality and to track data-related discrepancies for comparability over time or across countries. “The Demography of COVID-19 Deaths” database aims at bridging this gap. It provides COVID-19 death counts along with associated documentation, which includes the exact data sources and points out issues of quality and coverage of the data. The database — launched in April 2020 and continuously updated — contains daily cumulative death counts attributable to COVID-19 broken down by sex and age, place and date of occurrence of the death. Data and metadata undergo quality control checks prior to online release. As of mid-December 2021, it covers 21 countries in Europe and beyond. It is open access at a bilingual (English and French) website with content intended for expert users and non-specialists (https://dc-covid.site.ined.fr/en/; figshare: 10.6084/m9.figshare.c.5807027). Data and metadata are available for each country separately and pooled over all countries.

## Abstract (summary)

**Table:** 

Measurement(s)	mortality
Technology Type(s)	digital curation
Factor Type(s)	Death counts
Sample Characteristic - Organism	Homo sapiens • Covid-19 deaths
Sample Characteristic - Environment	Country
Sample Characteristic - Location	Austria, Belgium, Canada, England and Wales, Denmark, France, Germany, Italy, Japan, the Netherlands, Norway, Portugal, the Republic of Korea, the Republic of Moldova, Romania, Scotland, Spain, Sweden, Switzerland, Ukraine, and the United States of America

## Background & Summary

The COVID-19 pandemic represents an unprecedented health crisis. The importance of timely, high-quality, publicly accessible COVID-19 related data has been essential in order to monitor this impact. National authorities started collecting data, often through their health surveillance systems or by establishing *ad hoc* data collection systems after the start of the epidemic. Statistics on the number of COVID-19 cases, hospitalizations, intensive care admissions, and death counts, among other figures, became quickly available, often broken down at different geographical levels and for specific population characteristics. These data have been extensively recirculated, collated, commented on, and compared; however, they are usually poorly sourced and documented. Although there are other international databases of deaths related to COVID-19, some of which cover many countries (e.g. those compiled by the World Health Organization^1^, Our World in Data^2^, and John Hopkins University^3^), they all have limitations, mostly related to the quality and degree of detail in the metadata and lack of user-friendly ways to download time-series data. Because data on deaths attributable to COVID-19 are mainly imperfect statistics, the lack of documentation limits accurate comparisons.

“The Demography of COVID-19 Deaths” database^4^ focuses on collecting death counts attributable to COVID-19, providing details on the exact definition of COVID-19 death used in each country and the processing of the data. The database specifically aims to provide tools for assessing data coverage and comparability, over time and across countries that can be used by the research community and others (policymakers, journalists, etc.) for accurate trend analysis. Related documentation is collected from official statistics bureaus and epidemiological surveillance agencies, and published in the database with the following information:

1. Identify the exact source of data and its main features, such as the type of data collection system (e.g. health surveillance system, vital statistics, hospital records, etc.), collection and publication patterns, and quality control protocols.
2. Describe the conditions for reporting deaths from COVID-19 and assess the degree of completeness of the statistical information, as well as the changes over time in each country (e.g. whether the reported deaths only include those occurring in hospital settings or also those occurring in nursing homes or private residences).
3. Illustrate possible sources of misinterpretation, by drawing attention to the reference date for which deaths are reported and their possible reporting delays. Depending on the country, deaths may be reported at the time of occurrence, at the time of registration, at the time the information is entered into the statistical system, or at the time when the figures are officially published.
4. Specify the criteria considered in the attribution of a death to COVID-19 and its confirmation mechanisms. The criteria may vary among countries or between one data source and another within the same country. Some data sources only include confirmed cases, e.g. on the basis of biological tests or clinical diagnosis, while others also include suspected cases based on symptoms or proximity to a known case.

The database provides cumulative death counts by sex and age. This demographic information is an important determinant of COVID-19: older people are more vulnerable to the infection and its most severe forms, and differences between men and women have also been well-documented^5^. As such, variations in population structure are expected to affect the number of deaths and must be taken into account in international comparisons of COVID-19 mortality. The database also provides the most up-to-date population counts in each country. Information on the place and date of occurrence of the deaths was added where available.

Data collection started at the beginning of the pandemic. The database was launched on 2 April 2020. As of mid-December 2021, it covers 21 countries: Austria, Belgium, Canada, England and Wales, Denmark, France, Germany, Italy, Japan, the Netherlands, Norway, Portugal, the Republic of Korea, the Republic of Moldova, Romania, Scotland, Spain, Sweden, Switzerland, Ukraine, and the United States of America. An online survey on the database use revealed visits from researchers, students and journalists, with more than 660,000 views up to mid-December 2021.

The database was created and is distributed by the French Institute for Demographic Studies (INED by its French acronym), and will be maintained online even beyond the end the pandemic. INED has extensive experience in maintaining demography databases, through its involvement in databases such as the Human Mortality Database (mortality.org).

## Methods

The operational team of the “The Demography of COVID-19 Deaths” has selected the 21 countries in the database based on the availability of metadata and of periodic data publications. Each member of the database operational team collects death counts and the related documentation for up to two countries. These country specialists monitor data availability daily and collect all the information relating to COVID-19 mortality (statistical data and documentation) made available by national statistics offices and health institutions since the beginning of the pandemic. This implies that for each country, data are added retrospectively according to their date of reference (when possible). Country specialists prepare country data files in pre-established standard formats in Excel spreadsheets and update the country-specific documentation. For some countries data files can be formatted in statistical software because they are provided in .csv, .txt or .xlsx. However, for other countries this is not possible because the data comes in .pdf or in screen shots of online dashboard or images. For the latter countries, data must be copied “by hand” in the standard Excel spreadsheet.

Data for each country included in the database are accompanied by explanatory notes providing detailed explanations about data heterogeneity. These notes are structured in five sections and describe:

1. The data source(s), the specific institution and official website(s);
2. Data coverage (i.e. whether the data include only hospital deaths, all deaths, or deaths which occurred elsewhere, and, if available, a description of the cause-of-death certification process);
3. Data collection methods (i.e. information on the national protocols for reporting COVID-19 deaths);
4. The type of information originally available for each death (e.g. sex, age, date and place of occurrence, geography, comorbidity);
5. Publication frequency and data cut off time (i.e. the time at which the death count is stopped before publication by the organization in charge of disseminating the data);
6. A summary of any changes since the beginning of the pandemic in the criteria for the attribution of a given death to COVID-19 and in the data collection methods. The implementation dates of each of these changes are also indicated.

Until the beginning of July 2020, the data series were updated on a daily basis. Since then, the updates have been carried out on a weekly basis, as the pace of publication by the national statistics offices slowed down during the Summer of 2020.

The operational team completes the data update procedure by preparing and releasing the pooled data and metadata sets (i.e., files containing data for all countries combined). During this process the information collected for the countries is harmonized into categories that can be compared across data sources. Table 1 is an extract of the codebook of variables that describe the definition of a COVID-19 death for each data source in the database. The complete codebook can be downloaded at https://dc-covid.site.ined.fr/en/data/pooled-datafiles/ and in figshare^6^.

**Table 1:** Data definition variables.

Variables	Variable Modalities	Variable Description
COVID19 Definition	Confirmed COVID-19 deaths	Death of positive COVID-19 tested cases
	Confirmed and suspected COVID-19 deaths	Death of positive COVID-19 tested cases and suspected COVID-19 (symptoms were shown or/and contact with a positive COVID-19 tested was established) was likely and there was not laboratory confirmation
	Estimated COVID-19 deaths	Estimated COVID-19 deaths by a statistical model considering official imperfect statistics
Changes in the COVID19 definition	Yes	There are changes in the criteria used in the data series to attribute a given death to COVID-19
	No	There are not changes in the criteria used in the data series to attribute a given death to COVID-19. All published count undertook the same criteria.
COVID19_definition- changes		Detailed description of the changes and the date of implementation
Confirmation Mechanism	Positive PCR diagnosed by regional reference laboratories	Deaths of positive COVID-19 tested by PCR cases which diagnosis has been made by official laboratories, regardless of the causes of death
	Laboratory confirmation	Deaths of positive COVID-19 tested cases regardless of the causes of death
	Identified as cause of death + Laboratory confirmation	Deaths of positive COVID-19 tested cases in which COVID-19 has been identified as cause of death
	Identified as cause of death	Deaths with COVID-19 mention on the death certificate regardless of laboratory confirmation
	Underlying cause of death	Deaths with COVID-19 mention as underlying cause on the death certificate regardless of laboratory confirmation
Reference Date Type	Occurrence	The date of reference is the date of occurrence of the death
	Registration	The date of reference is the date of notification to the civil registry
	Report	The date of reference is the date of notification to the health agencies/ Surveillance Systems
Reference date unit	Day	Exact day of reference
	Week	The week ending date of reference
Place Coverage	Hospital	COVID–19 deaths occurred or reported in hospitals, or within the public health system.
	Hospital & hospice	COVID–19 deaths occurred in hospitals and hospice facilities (e.g. Care homes, EPHAD, long-term care facilities)
	All places	COVID–19 deaths, whether the death took place in a hospital, in an institution or at home (i.e., all places of death)

## Data Records

“The Demography of COVID-19 Deaths” is freely available online [https://dc-covid.site.ined.fr/en/] for scientific use (see “Terms of use” page on the website: https://dc-covid.site.ined.fr/en/contact-terms-use/). A snapshot of the dataset is also available at the figshare repository^6^. The database aims at addressing the needs and interests of a variety of database users, including researchers as well as non-specialists and the general public. The database consists of a collection of webpages: one for each country, one for the pooled datasets, and additional webpages including a discussion of key data issues.

In the *country-specific webpages* [https://dc-covid.site.ined.fr/en/data/], for each country, the following files are available for download: 1) a spreadsheet file with the actual data coming from one or (if applicable) several data sources, also containing a summary of the metadata on each data source, 2) a document containing country-specific explanatory notes, and 3) all the original documentation (methodological documents and official reports) from which the data and the metadata have been extracted.

**Fig. 1:**
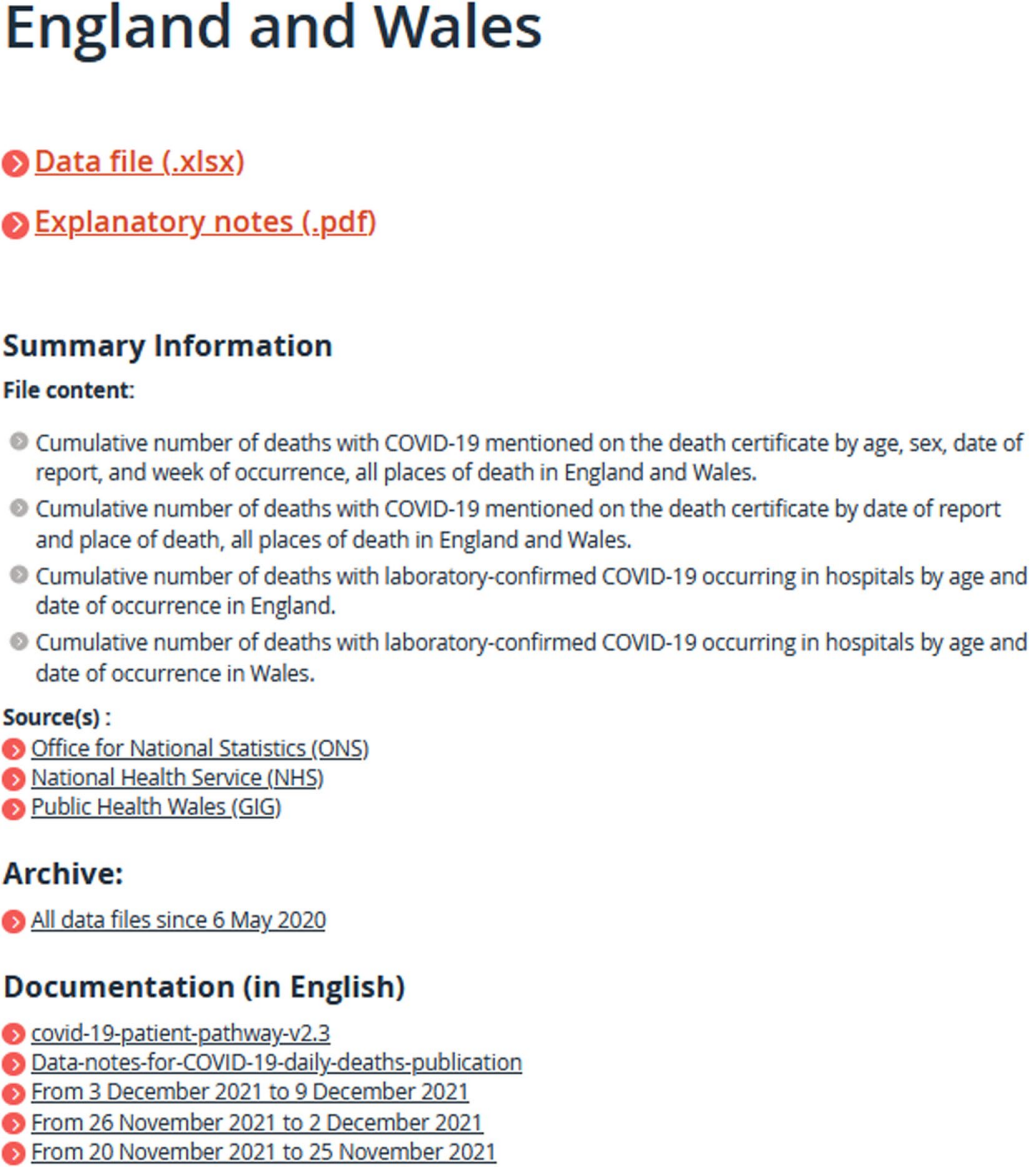
Screen shot of a typical country page.

The original documentation can be used to specify data characteristics, data heterogeneity, and possible biases across different sources, for rigorous international comparisons. Each country-specific webpage also displays some summary information about coverage and the national sources of information, as well as a link to the archive containing all prior versions of the data files (Fig. 1). The country-specific webpage is intended for non-specialist users. It provides data in a user-friendly format along with warnings about data idiosyncrasies that may hamper country comparison.

On the *Pooled sets* webpage [https://dc-covid.site.ined.fr/en/data/pooled-datafiles/], pooled data sets are available for expert users in .csv format. These data sets include all the data sources except for those that have been discontinued. They can be downloaded in a zipped folder along with a file containing population estimates, the pooled metadata set, and a codebook of variables. Countries in the pooled data sets are referenced with the ISO 3166 numeric and alpha-3 codes which helps to geolocate and merge the data with indicators available in other international databases such as testing, short-term estimates of excess mortality, vaccination, and policy indicators.

The following zipped folders are available:

1. Cumulative deaths by sex and age https://www.ined.fr/fichier/rte/166/Page%20Data/Pooled%20Datasets/AgeSex.zip],
2. Cumulative deaths by places of death [https://www.ined.fr/fichier/rte/166/Page%20Data/Pooled%20Datasets/PlaceOfDeaths.zip],
3. Cumulative deaths by publication dates [https://www.ined.fr/fichier/rte/166/Page%20Data/Pooled%20Datasets/PublicationDate.zip],
4. Cumulative deaths by occurrence date of the deaths [https://www.ined.fr/fichier/rte/166/Page%20Data/Pooled%20Datasets/OccurenceDate2021.zip].

The pooled data sets can be downloaded from the website, including previous versions of the data files. They are also available in figshare^6^ updated as of mid-December 2021, along with an R code that can be used to retrieve the latest version of these datasets from the website.

The pooled metadata set summaries the main characteristics of the data for proper international comparisons, such as the definition of deaths attributable to COVID-19, confirmation criteria, data coverage, co-variables, and the collection and publication patterns of each data source (Tables 1 and 2). Metadata for the population estimates are also available. Through the R code available in figshare^6^, users can merge data and metadata pooled files to keep track simultaneously of changes in the data and in the definitions over time. Merged data and metadata pooled files can be used to select comparable subsets according to data characteristics.

**Table 2:** Description of variables contained in the pooled metadata set and in the online *Data Availability Explorer* about the death counts and data collection methods.

Variables	Variable Modalities	Variable Description
Sex	Age and Sex combined	Death counts broken down by sex
	Total sex	Death counts broken down by sex without specific ages
	Not	The information available does not have disaggregation by sex
Age Groups Length	5-year intervals	Death counts broken down by 5-year age groups
	10-year intervals	Death counts broken down by 10-year age groups
	20-year intervals	Death counts broken down by 20-year age groups
	Not	The information available does not have age group
Age Group Distribution	Same range size	All age groups have the same length, even interval sizes
	Different range size	Age groups have difference lengths, uneven interval sizes
	Not	The information available does not have age group
Ending Age Group	80+	The ending-age group closes at population aged 80 years and over
	85+	The ending-age group closes at population aged 85 years and over
	90+	The ending-age group closes at population aged 90 years and over
	95+	The ending-age group closes at population aged 95 years and over
	100+	The ending-age group closes at population aged 100 years and over
	Not	The information available does not have age group
Place of Death	Hospital	Healthcare setting, inpatient Healthcare setting, outpatient or emergency room, Healthcare setting dead on arrival
	Care Home	Hospice, Nursing home/long term care facility, Care homes
	Home	Decedent’s home, Private home
	Elsewhere	Other places than Hospital and Hospice
	Unknown	Cases with unknown information on place of death
	Not	The information available does not have place of death
First Date	DD-MM-YYYY	First date of reference in this database
Completeness	Complete	The given information represents the total number of deaths being publicly available by all national authorities
	Incomplete	The given information represents a portion of the total number of deaths being publicly available by all national authorities
Collection System	Surveillance System	Information coming from the Surveillance System under the authority of the National Ministry of Health
	Vital statistics system	Information coming from the Civil Registration and Vital Statistics System
	Transmission from local authorities and hospitals	Information collected from the centralized report of local authorities, prefectures or regional health systems
	Daily electronic transmission via Civil Protection Bulletin	Information collected by Civil Protection
Countdown	Daily	Cumulative death counts refer to daily reports
	Every two days	Cumulative death counts refer to reports made every two days
	Twice a week	Cumulative death counts refer to reports made twice a week
	Weekly	Cumulative death counts refer to weekly reports
Countdown Stop Time	HH	Time in which figures stopped being count regardless the time and date of their publication
Reference-Publication Lag	Same day	Countdown stop time and publication have the same date
	Previous day	Countdown stop time is one day before the publication
	Between 1 to 4 days	Countdown stop time is between 1 and 4 days before the publication
	More than 10 days	Countdown stop time is more than 10 days before the publication
Time	hh/DAY /	Time in which information is published by the official sources regardless of the countdown stop time
Format	xlsx	The information is extracted from official sources in excel format
	csv	The information is extracted from official sources in csv format
	PDF	The information is extracted from official sources in PDF format
	Website	The information is extracted from the official sources dashboard as an image file

Users can explore the data availability in the database according to metadata characteristics through an online *Data availability explorer* [https://ineddemographiecovid19.shinyapps.io/DataViz/]. This tool shows the heterogeneity of the data across data sources and over time, as to e.g.: width of age groups, data collection systems, data coverage, identification of COVID-19 (suspected or confirmed, cause of death), reference date (e.g., registration, publication, or occurrence date). It can be used to identify data sources with similar characteristics, which can therefore be compared. Because some data sources have changed their characteristics over time, sound comparisons could be limited to specific periods. The explorer is updated monthly.

## Technical Validation

Collected data undergo a number of validations before and after publication. With the aim of ensuring that both the data and the associated documentation are of the highest quality, before the data for a new country are published in the database, an in-country expert is contacted to contextualize and interpret the information. The national expert provides feedbacks about the most reliable data sources and checks the accuracy of the explanatory notes prepared by the database team. In addition, three team members oversee the work carried out by the country specialists before the publication of each country update. They check the accuracy of the daily data against the original data sources and monitor completeness of the documentation. This is especially important for those countries for which the data have to be copied manually because the data are only available in formats that are not reusable in statistical software (e.g. pdf. and online dashboard or images).

The database team also performs other types of validations through data analyses. The first one is the graphical representations of the weekly standardized death rates since the beginning of the pandemic in each country. These graphs are available online for illustrative purposes [https://www.ined.fr/fichier/rte/166/Page%20accueil/Taux-eng.jpg] and can be used to review the long-term trends of the pandemic. In addition, the team analyzed the COVID-19 data-related issues that may hinder international comparisons in a published scientific paper^5^. During this analysis, the operational team conducted comparisons with other datasets, such as the Human Mortality Database (HMD). Specifically, the age-and-sex proportional distributions of the cumulative number of COVID-19 deaths having occurred during the first wave (deaths occurred up to September 15, 2020) were assessed using the age-and-sex distributions of previous all-cause mortality reported by the HMD. Further analysis considered comparisons among countries based on classic demographic indicators such as sex ratios of age-standardized and age-mortality patterns. The database has also been used by international researchers and has already led to articles published in influential journals such as *The Lancet*^7^, *Proceedings of the National Academy of Science*^8,9^, and *Demographic Research*^10^.

The stringency of the data collection and metadata review processes has limited the number of countries available in the database. In some cases, there may be no country specialist and/or in-country expert in the database operational team to ensure that the data available are properly interpreted. In other cases, national statistics offices in charge of disseminating COVID-19 mortality data do not provide documentation that is detailed enough to assess the quality and comparability of the available statistical information. These are important criteria for a country to be included in the database.

## Usage Notes

“The Demography of COVID-19 Deaths” database is an open access database [https://dc-covid.site.ined.fr/en/]. No registration is required. Anyone can browse the database content and download the data, which are available both as spreadsheets and .csv files. Specific citation guidelines are indicated in a “Terms of use” page on the website. Users are invited to contact the database team at eo-dc-covid@listes.ined.fr with their questions or suggestions, or to volunteer their help with accessing data from additional countries.

In addition to providing data to users, the “The Demography of COVID-19 Deaths” database highlights the potential uses that may be made of the data, while pointing out the specificities, differences and shortcomings of the data sets [https://dc-covid.site.ined.fr/en/presentation]. In this sense, a core feature of the database is to illustrate critical data issues to be considered when conducting comparisons over time and/or across countries. Simple cross-country examples are given to illustrate these issues and demonstrate the need to take them into consideration when analyzing pandemic mortality data.

**Fig. 2:**
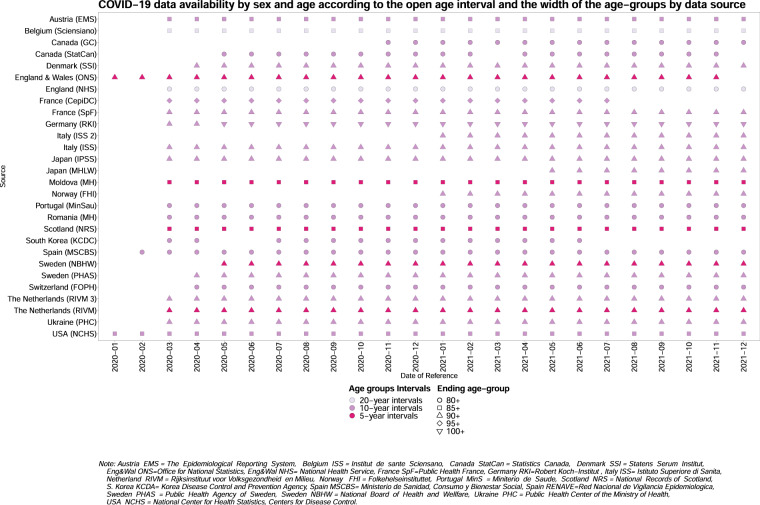
COVID-19 data availability by sex and age according to the open age interval and the width of the age-groups by data source. This figure was created through the *Data Availability Explorer* here https://ineddemographiecovid19.shinyapps.io/DataViz/. Only one date per month is shown, but the same figure for all the available dates can be visualized online.

**Fig. 3:**
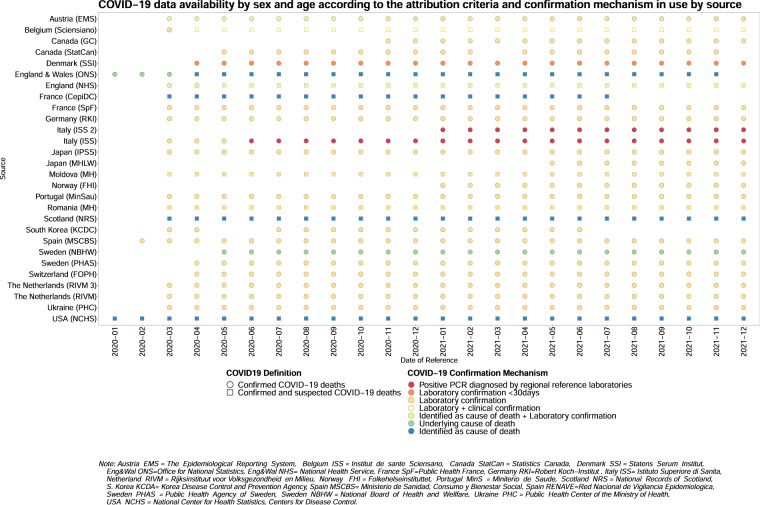
COVID-19 data availability by sex and age according to the attribution criteria and confirmation mechanism in use by each source. This figure was created through the *Data Availability Explorer* here https://ineddemographiecovid19.shinyapps.io/DataViz/. Only one date per month is shown, but the same figure for all the available dates can be visualized online.

Users are also invited to check the *Data availability explorer* before engaging in international comparison. The database team’s first analysis pointed out the need to take data heterogeneity into account when identifying the national and transnational characteristics of COVID-19 mortality rates and trends^5^. The availability and degree of detail of the COVID-19 death counts by age and sex varies between data sources. Variations range from differences in the open-ended age interval (the maximum age to which deaths are reported) and the age-group intervals (Fig. 2), to the diversity of the mechanisms implemented for confirming COVID-19 infections or attributing a death to COVID-19 (Fig. 3), all of which may bias comparison. Regarding the latter point, while most data sources rely on confirmation of COVID-19 through laboratory testing or clinical symptoms, some accept more loosely defined criteria and include suspected as well as confirmed COVID-19 deaths. Users can harmonize age-group intervals at their convenience; however, they cannot do anything about the other sources of heterogeneity in the data. To correctly interpret the pattern of trends and international differences in COVID-19 pandemics, therefore, comparisons should only be carried out between sources that use comparable COVID-19 attribution and confirmation methods^5^.

Similarly, data sources vary according to the type of collection system, though surveillance systems (i.e. systems put in place by governments in urgent situations) are the most common. The type of data collection system affects the process of data preparation and updating, as well as the frequency with which data are released. Surveillance systems and systems based on the transmission of information from local authorities or hospitals to the centralized body are often associated with the daily release of updated data. Systems based on the vital statistics system, on the other hand, tend to release data on a less frequent basis because the data undergo verification protocols and corrections. These differences in data collection system types and data release procedures may affect the degree of completeness of the data: the faster the data is released, the less accurate they are likely to be.

The “Demography of COVID-19 Deaths” database is the only database so far to collect, update, and systematize the metadata associated with the mortality attributed to COVID-19. Detailed metadata enable users to make informed decisions regarding the most suitable data for their comparative analyses and to understand the main limitations of the results. This is a major strength of the database because most reported COVID-19 statistics come from *ad hoc* systems. Its user-friendly structure helps users with varying levels of expertise to find the information collected at any time, as it provides continuous access to previously released files and official documentation for each country.

## Data Availability

Along with the pooled data and metadata files as of mid-December 2021, users can find in figshare^6^ an R script to retrieve the latest version of the pooled data and metadata files and update them. This script can also be used to merge the pooled data and metadata files.

## References

[1] WHO Coronavirus *(COVID-19) Dashboard* https://covid19.who.int (2021).

[2] Ritchie, H. *et al*. *Coronavirus Pandemic (COVID-19) Our World in Data* https://ourworldindata.org/coronavirus (2020).

[3] Dong E, Du H, Gardner L (2020). An interactive web-based dashboard to track COVID-19 in real time. Lancet Infect. Dis..

[4] The Demography of COVID-19 Deaths. *French Institute for Demographic Studies (INED)* https://dc-covid.site.ined.fr/en/ (2021).

[5] Garcia J (2021). Differences in COVID-19 mortality: Implications of imperfect and diverse data collection systems. Population.

[6] Caporali A (2022). figshare.

[7] Bhopal SS, Bhopal R (2020). Sex differential in COVID-19 mortality varies markedly by age. The Lancet.

[8] Goldstein, J. R., Cassidy, T. & Wachter, K. W. Vaccinating the oldest against COVID-19 saves both the most lives and most years of life. *Proc. Natl. Acad. Sci*. **118** (2021). 10.1073/pnas.2026322118 PMC7980436 33632802

[9] Goldstein JR, Lee RD (2020). Demographic perspectives on the mortality of COVID-19 and other epidemics. Proc. Natl. Acad. Sci..

[10] Sasson I (2021). Age and COVID-19 mortality: A comparison of Gompertz doubling time across countries and causes of death. Demogr. Res..

